# Detection of *ROS1* gene fusions using next-generation sequencing for patients with malignancy in China

**DOI:** 10.3389/fcell.2022.1035033

**Published:** 2022-12-15

**Authors:** Ning Li, Zhiqin Chen, Mei Huang, Ding Zhang, Mengna Hu, Feng Jiao, Ming Quan

**Affiliations:** ^1^ Department of Oncology, Shanghai General Hospital, Shanghai Jiao Tong University School of Medicine, Shanghai, China; ^2^ Department of Oncology, Shanghai East Hospital, School of Medicine, Tongji University, Shanghai, China; ^3^ Department of Oncology, Yancheng Third People’s Hospital, Yancheng, China; ^4^ The Medical Department, 3D Medicines Co., Ltd., Shanghai, China; ^5^ Department of Oncology, Renji Hospital, School of Medicine, Shanghai Jiao Tong University, Shanghai, China

**Keywords:** solid tumor, next-generation sequencing, ROS1 fusions partners, ROS1 breakpoint, lung cancer

## Abstract

**Objective:** This study aimed to identify *ROS1* fusion partners in Chinese patients with solid tumors.

**Methods:** Next-generation sequencing (NGS) analysis was used to detect *ROS1* rearrangement in 45,438 Chinese patients with solid tumors between 2015 and 2020, and the clinical characteristics and genetic features of gene fusion were evaluated. H&E staining of the excised tumor tissues was conducted. Samples with a tumor cell content ≥ 20% were included for subsequent DNA extraction and sequencing analysis.

**Results:** A total of 92 patients with *ROS1* rearrangements were identified using next-generation sequencing, and the most common histological type lung cancer. From the 92 *ROS1* fusion cases, 24 *ROS1* fusion partners had been identified, including 14 novel partners and 10 reported partners. Of these, *CD74*, *EZR*, *SDC4*, and *TPM3* were the four most frequently occurring partners. Fourteen novel *ROS1* fusion partners were detected in 16 patients, including *DCBLD1-ROS1*, *FRK-ROS1*, and *VGLL2-ROS1*. In many patients, the *ROS1* breakpoint was located between exons 32 and 34.

**Conclusion:** This study describes 14 novel *ROS1* fusion partners based on the largest *ROS1* fusion cohort, and the *ROS1* breakpoint was mostly located between exons 32 and 34. Additionally, next-generation sequencing is an optional method for identifying novel *ROS1* fusions.

## Highlights

1. This study detected *ROS1* fusion partners and the *ROS1* fusion breakpoint in solid tumors of Chinese patients in the largest *ROS1* fusion cohort to date.
2. A total of 14 novel *ROS1* fusions in solid tumor of Chinese patients were identified.
3. The majority of patients had a *ROS1* breakpoint between exons 32 and 34.

## Introduction

Several gene-targeting therapies have been developed to treat human malignancies. With an in-depth study of the biological mechanism of tumor cells, the status of target genes in gene targeting therapy, such as *EGFR*, *MET*, and *ROS1*, has gradually become prominent (Cao et al., 2020). ROS1 is a receptor tyrosine kinase whose activation is reported to be linked to the growth and proliferation of malignant tumors. Previous studies have reported that *ROS1* undergoes gene rearrangement in many malignant tumors, such as lung cancer and liver cancer. In addition, *ROS1* rearrangements rarely overlap with alterations in EGFR, KRAS, or other targeted oncogenes (Zhu et al., 2018). *ROS1* was originally identified in 1986 as a viral proto-oncogene with unique oncogenic effects in the UR2 avian sarcoma virus. Hybridization analysis revealed that *ROS1* was located in the human chromosome region 6ql6–q22 and was further positioned on chromosome 6q22.1 (Birchmeier et al., 1986; Matsushime et al., 1986).

*ROS1* gene fusion expression can drive cell proliferation and induce malignant transformation, which is common in many tumor cells, such as malignant gliomas (Sievers et al., 2021). ROS1 fusions occur in 1%–2% of NSCLC, and the prevalence is higher among patients in Asian countries (including China) than in Western countries (Cai and Su, 2013; Davies and Doebele, 2013; Melosky et al., 2021). A synthetic study has introduced the role of *ROS1* in various cancers. It has been reported that 26 genes fuse with *ROS1*. With the advances in sequencing technology, many new genes have been reported to fuse with *ROS1*. Natural *ROS1* rearrangement was first found in the human brain glioblastoma cell line U118MG (Birchmeier et al., 1987; Charest et al., 2003). The deletion of chromosome six led to the fusion of the *ROS1* gene into the *FIG* gene, which was observed in samples from patients with hepatobiliary carcinoma and ovarian cancer (Birch et al., 2011; Suehara et al., 2012). Numerous studies have shown that crizotinib achieves good results in NSCLC patients with positive *ROS1* rearrangement (Shaw et al., 2011; Camidge et al., 2012). AP26113, a specific inhibitor of *ROS1*, exhibits additional inhibitory activity against oncogenic *ROS1* fusions that are involved in the clinical treatment of patients with advanced solid tumors (Anjum et al., 2012). Thus, it is important to detect and evaluate *ROS1* rearrangements and gene fusion in patients with malignancies.

Currently, there are many methods for detecting *ROS1* fusion genes, including qRT-PCR, FISH, IHC, and NGS (Sakai et al., 2019). These methods have advantages and drawbacks. For example, owing to the continuous increase in *ROS1* gene fusion partners, some positive cases may be missed by RT-PCR (Shaw et al., 2011). Fluorescence *in situ* hybridization (FISH) requires advanced technology, which limits its use. Immunohistochemistry (IHC) is suitable for screening existing fusion expression, whereas only NGS can detect novel *ROS1* fusion partners (Zhang et al., 2019). More than 30 *ROS1* fusion gene partners have been reported in lung cancer, glioma, and hepatic angiosarcoma, containing *CD74*, *SLC34A2*, *GOPC* (Zhu et al., 2019). NGS is regarded as a powerful tool for detecting *ROS1* rearrangements because of its accuracy, sensitivity, and specificity.

Although some studies have reported fusion partners of *ROS1*, more novel fusions are still being reported. In addition, the distribution of *ROS1* fusions varies among different types of cancer. Exploring *ROS1* fusions in different cancers may help identify more precise therapies. Therefore, this study aimed to detect the *ROS1* partners of Chinese patients with solid tumors using NGS. This study may offer a novel understanding of the treatment of solid tumors based on the *ROS1* fusion profile.

## Material and methods

### Patient information

A total of 45,438 Chinese patients with solid tumor were treated in the 3Dmed Lab (Shanghai, China) between 2015 and 2020. *ROS1* gene fusion was confirmed by NGS. This study was approved by Shanghai East Hospital, Tongji University School of Medicine (No. 2020-093).

### Sample collection and DNA extraction

Pathological results were used to screen the samples for subsequent analysis. Hematoxylin and eosin (H&E) staining of the removed tumor tissues was conducted. Samples with tumor cell content ≥ 20% were included for subsequent analysis. Genomic DNA (gDNA) was extracted using the ReliaPrep™ FFPE gDNA Miniprep System (Promega) and quantified using a Qubit™ dsDNA HS Assay Kit (Thermo Fisher Scientific). The cfDNA in plasma was extracted using the QIAamp Circulating Nucleic Acid Kit (Qiagen), and the gDNA in white blood cells was extracted using the QIAamp DNA Mini Kit (Qiagen).

### Library preparation and targeted capture

Sequencing libraries were established as described previously (Shu et al., 2017). Probe-based hybridization was carried out on the index library with a custom NGS panel, in which the probe bait was a single compound 5 biotinylated 120 bp DNA oligonucleotide. All these contain introns of *ROS1* for fusion detection. Repetitive elements were screened and removed from the baits of introns, as previously described (Karolchik et al., 2004).

### DNA sequencing and data processing

The extracted DNA was analyzed using a NovaSeq 6,000 platform (Illumina) to screen for targeted gene rearrangements (Xia et al., 2020). The detection approach for variants was based on a binomial test, and an R package was developed to analyze these variants through the analysis of unique supporting read depth, strand bias, and base quality (Su et al., 2017). The variants were analyzed using an automatic false-positive filtering pipeline to ensure specificity and sensitivity when the allele frequency was ≥ 5%. ANNOVAR was performed against dbSNP (v138), 1000Genome, and ESP6500 to annotate the SNPs, insertions, and deletions. Only missense, stop-gain, frameshift, and non-frameshift indel mutations were retained for gene rearrangement analysis.

## Results

### Patient characteristics

**FIGURE 1:**
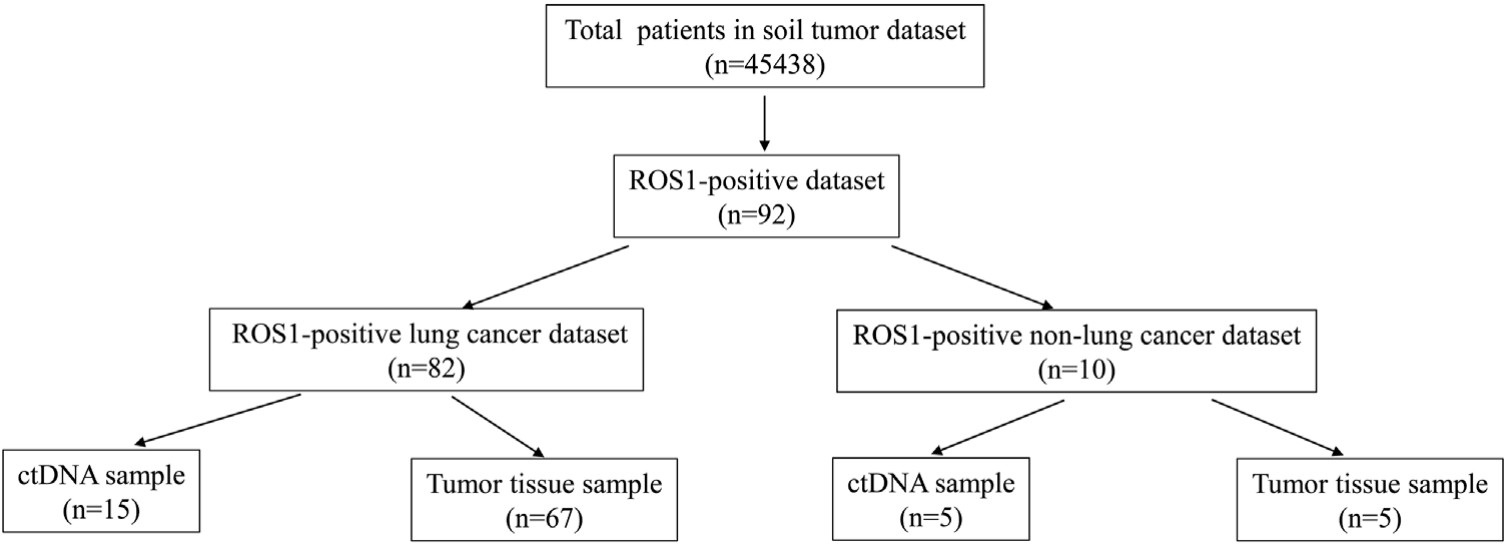
Study population. Schematic representing the population of patients in this study.

A total of 45,438 solid tumor patients from the 3Dmed lab were evaluated, of which 92 patients were identified to have *ROS1* rearrangements in the blood or tumor tissues (Figure 1). The basic features of the 92 patients were analyzed. Their average age was 57 years (range, 16–82 years) and 63.0% (58/92) of the patients were female (Table 1). As shown in Supplementary Table S1, there were 82 cases of lung cancer, two cases of gastric cancer, two cases of retroperitoneal neoplasm, and one case of liver cancer, epithelioid hemangioendothelioma, liposarcoma, schwannoma, colorectal cancer, and squamous cell carcinoma. Lung adenocarcinoma was diagnosed in 78.9% (68/92) of the patients (Table 1).

**TABLE 1:** Clinicopathologic features of patients with *ROS1* rearrangement (*n* = 92).

	Total (*n* = 92)	Lung cancer (*n* = 82)	CD74-ROS1 lung cancer (*n* = 35)	Non-CD74-ROS1 lung cancer (*n* = 47)
Age				
≥60	41	37	12	25
<60	51	45	23	22
Sex				
Male	34	27	13	14
Female	58	55	22	33
Histological types				
Lung adenocarcinoma	68	68	26	42
Lung Squamous Carcinoma	1	1	1	0
NA	23	13	8	5
TMB				
NA	52	46	20	26
≥10	6	6	3	3
<10	34	30	12	18
TPS				
NA	60	54	23	31
<1%	9	7	2	5
1%–49%	16	14	8	6
≥50%	7	7	2	5
CPS				
NA	74	67	29	38
<1%	2	2	0	2
1%–49%	14	11	6	5
≥50%	2	2	0	2

### *ROS1* fusion partners

**FIGURE 2:**
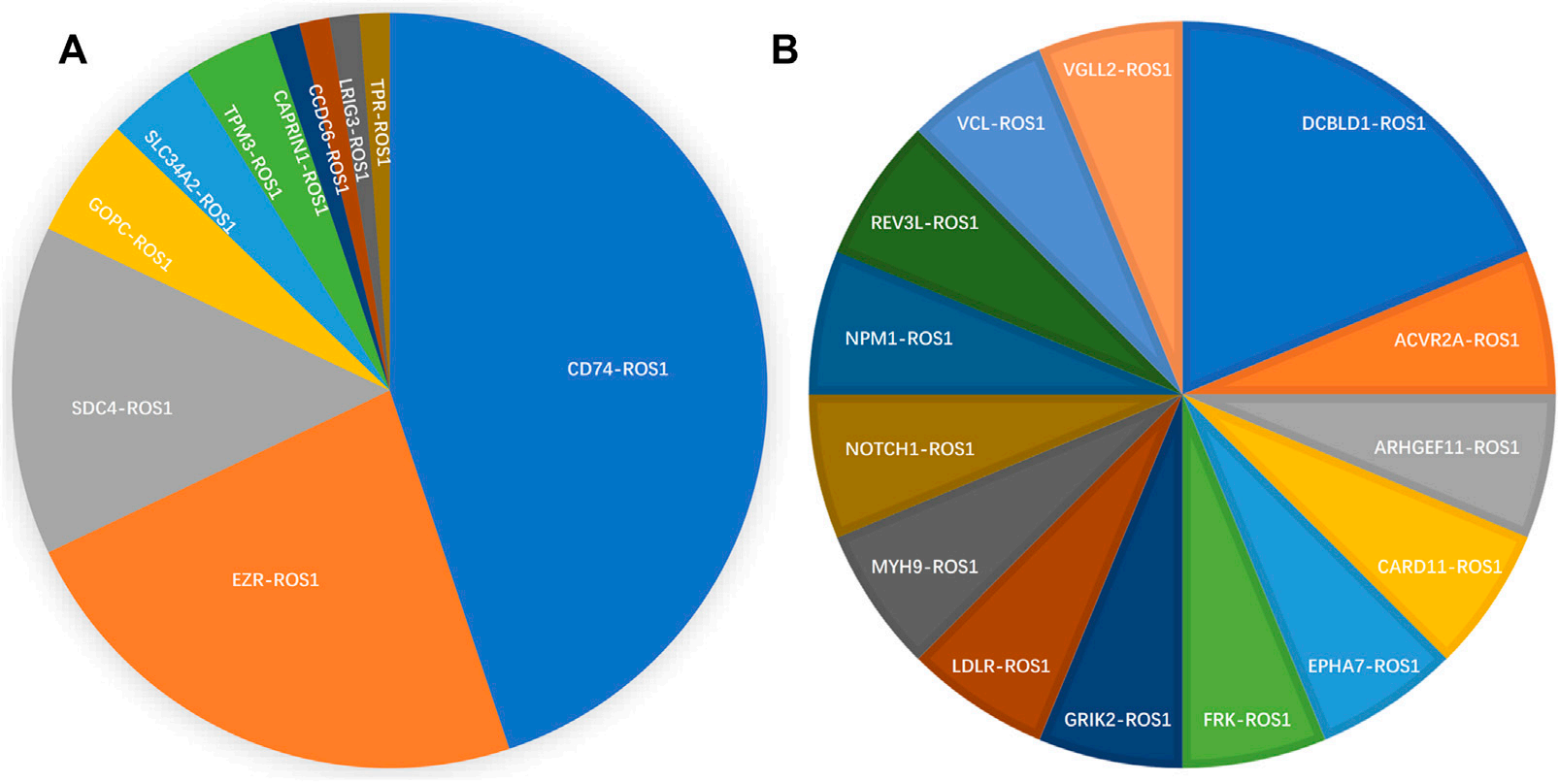
Spectrum of *ROS1* fusion partners. **(A)** 10 reported *ROS1* fusion partners. **(B)** 14 novel *ROS1* fusion partners.

From 92 *ROS1* fusion cases, 24 *ROS1* fusion partners were identified, including 14 novel partners and 10 reported partners. Cluster of differentiation 74 (*CD74*), ezrin (*EZR*), syndecan 4 (*SDC4*), and tropomyosin 3 (*TPM3*) were the four most frequently occurring fusion partners. The reported *ROS1* fusion partners were identified in 75 cases (81.5%, 75/92) and their distribution was as follows: *CD74-ROS1* (38.0%, 35/92), *EZR-ROS1* (19.6%, 18/92), *SDC4-ROS1* (12.0%, 11/92), *GOPC-ROS1* (4.3%, 4/92), *SLC34A2-ROS1* (3.2%, 3/92), *TPM3-ROS1* (3.2%, 3/92), *CAPRIN1-ROS1* (1.1%, 1/92), *CCDC6-ROS1*(1.1%, 1/92), *LRIG3-ROS1*(1.1%, 1/92), and *TPR-ROS1* (1.1%, 1/92) (Figure 2A).

Fourteen novel *ROS1* fusion partners were identified in the 16 patients. Of these, novel *DCBLD1-ROS1* was observed three times in three patients with lung cancer, and other novel fusion partners were observed only once in six lung cancer cases. Moreover, seven novel partners were identified in two retroperitoneal neoplasm patients (*FRK-ROS1* and *VGLL2-ROS1*), one gastric cancer patient (*ARHGEF11-ROS1*), one liver cancer patient (*REV3L-ROS1*), one liposarcoma patient (*EPHA7-ROS1*), one patient with epithelioid hemangioendothelioma (*NOTCH1-ROS1*), and one squamous cell carcinoma patient (*CARD11-ROS1*). In addition, two patients with lung cancer harbored two *ROS1* fusions (Figure 2B). One patient had *CD74-ROS1* and *SLC34A2-ROS1* fusions, and the other patient had novel *DCBLD1-ROS1* and *GOPC-ROS1* fusions.

### *ROS1* fusion breakpoints

**FIGURE 3:**
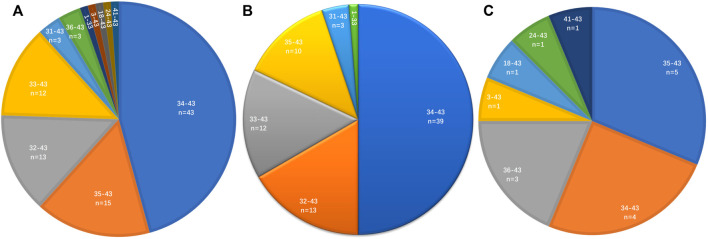
Distribution of *ROS1* breakpoints. **(A)** *ROS1* breakpoints of *ROS1* fusion patients. **(B)** *ROS1* breakpoints of reported *ROS1* fusion types. **(C)** *ROS1* breakpoints of novel *ROS1* fusion types in patients.

The distribution of *ROS1* fusion partners and *ROS1* breakpoints was investigated. The results showed that the *ROS1* breakpoint was mostly located between exons 32 and 34, which was more apparently in reported *ROS1* fusion types (Figures 3A–C), without affecting the transmembrane and tyrosine kinase domains of the ROS1 protein. A breakpoint at exon 35 was found in 15 patients, which disrupted the transmembrane domain (Tables 2, 3).

**TABLE 2:** ROS1 fusion variants described in reported *ROS1* fusion patients.

Reported partner	Number	5′ gene exon	Number	3′ gene exon	Number
CD74-ROS1	35	1–6	16	34–43	15
				33–43	1
		1–4	15	34–43	12
				33–43	2
				32–43	1
		1–2	1	34–43	1
		1–7	1	34–43	1
		1–8	1	33–43	1
		7–8	1	34–43	1
EZR-ROS1	18	1–9	17	32–43	7
				33–43	7
				32–43	2
				31–43	1
		1–10	1	34–43	1
SDC4-ROS1	11	1–2	8	32–43	8
		1–4	2	32–43	2
		1–5	1	34–43	1
GOPC-ROS1	4	1–8	3	35–43	3
		1–4	1	36–43	1
SLC34A2-ROS1	3	1–12	1	1–33	1
		1–13	1	33–43	1
		1–4	1	31–43	1
TPM3-ROS1	3	1–10	2	31–43	1
				35–43	1
		1–7	1	35–43	1
CAPRIN1-ROS1	1	1–7	1	35–43	1
CCDC6-ROS1	1	1–5	1	35–43	1
LRIG3-ROS1	1	1–17	1	35–43	1
TPR-ROS1	1	1–4	1	35–43	1

**TABLE 3:** ROS1 fusion variants described in novel *ROS1* fusion patients.

Novel partner	Number	5′ gene exon	Number	3′ gene exon	Number
DCBLD1-ROS1	3	1–14	1	35–43	1
		15–15	1	35–43	1
		1–9	1	35–43	1
ACVR2A-ROS1	1	6–12	1	41–43	1
ARHGEF11-ROS1	1	1–41	1	34–43	1
CARD11-ROS1	1	26–25	1	3–43	1
EPHA7-ROS1	1	6–17	1	36–53	1
FRK-ROS1	1	1–7	1	18–43	1
GRIK2-ROS1	1	1–10	1	34–43	1
LDLR-ROS1	1	1–14	1	34–43	1
MYH9-ROS1	1	1–40	1	35–43	1
NOTCH1-ROS1	1	1–30	1	34–43	1
NPM1-ROS1	1	1–4	1	35–43	1
REV3L-ROS1	1	23–33	1	24–43	1
VCL-ROS1	1	1–16	1	36–43	1
VGLL2-ROS1	1	4–4	1	36–43	1

## Discussion

Gene fusion is an ideal target for cancer therapy; therefore, reliable detection of gene fusion is necessary. Many methods have been developed for detecting gene fusion, such as FISH, IHC, qRT-PCR, and NGS, which have different characteristics and applications. In clinical practice, FISH is considered the gold standard for detecting *ROS1* fusions (Chiari et al., 2020; Shen et al., 2020). Since FISH is a confirmation test, it may be applied to detect existing *ROS1* fusions with high accuracy. However, it is challenging to identify novel ROS1 partners using FISH and simultaneous detection of *ROS1* fusion is limited. Due to this facile manipulation, IHC seems to be user-friendly, cost-effective, and highly sensitive. However, it is mostly a complementary tool to other methods, since the results should be observed and analyzed by skilled personnel. Similar to FISH and IHC, qRT-PCR can be used to detect reported fusions, and it exhibits better throughput, sensitivity, and specificity. NGS is an emerging tool for screening *ROS1* fusions. A unique advantage of NGS is that it is an innovative and optimal assay for identifying novel fusions. Woo et al. detected glioblastomas harboring gene fusions using NGS in 356 diffuse gliomas. They identified 53 patients harboring various oncogenic gene fusions, including MET, EGFR, and FGFR. They also identified two patients with novel CCDC6-RET fusions (Woo et al., 2020). NGS is a promising approach for obtaining the distribution information of fusions. Thus, our study also used NGS fusion assays to detect the profiles of *ROS1* rearrangement in 45,438 patients with malignancies in China, which is the largest *ROS1* fusion cohort screened to date, which led to reliable results. It is important to screen for new *ROS1* fusions, which can be further confirmed and applied as therapeutic targets.

Another advantage of NGS for detecting *ROS1* fusion is that blood samples can be used, including plasma and white blood cells. In this study, positive *ROS1* fusion was observed in the blood samples of patients with lung cancer (*n* = 15) and other cancers (*n* = 5). This indicated that *ROS1* fusions could also be detected in plasma samples, even for the detection of novel fusion types, which was the same as that in tissue samples.

*ROS1* fusions were identified in 92 patients. A total of 10 reported and 14 novel *ROS1* fusions were found in the solid tumors of Chinese patients with various malignancies. *CD74*, *EZR*, *SDC4*, and *TPM3* were 4 most frequently occurring fusion partners. Matsuura et al. (2013) detected common fusion genes in 114 NSCLCs using RT-PCR. They found that the *CD74-ROS1* fusion was involved in the carcinogenesis of a subpopulation of NSCLC, which may assist in clarifying the features of tumors and guiding treatment. The *CD74-ROS1* fusion gene was reported for the first time in an inflammatory breast cancer patient by NGS (Hu et al., 2021). Cui et al. found that *CD74-ROS1* was the most frequently occurring fusion protein in NSCLC (Cui et al., 2020), which is in line with the results of this study. Tatjana et al. (2022) first reported the *EZR-ROS1* fusion identified in renal cell carcinoma by molecular sequencing. This new fusion was significant, as crizotinib may be effective in future treatment. The existing *ROS1* fusions can be detected in samples from patients, and the positive results may provide information for subsequent therapies, such as the use of kinase inhibitors.

Different *ROS1* fusions were observed in one case. Our results indicated that two patients with lung cancer harbored two *ROS1* fusions. One patient had *CD74-ROS1* and *SLC34A2-ROS1* fusions, and the other patient had novel *DCBLD1-ROS1* and *GOPC-ROS1* fusions. This finding has also been reported in previous studies. Using NGS, Xu et al. found two *ROS1* fusions [*SDC4-ROS1* (EX2:EX32) and *ROS1-GK* (EX31:EX13)] in a patient with lung adenocarcinoma. These results indicated that the patient might be sensitive to *ROS1* inhibitors (Xu et al., 2021). Patients harboring *ROS1* fusions may benefit from crizotinib if their tumors are metastatic (Cai et al., 2019).

With the large number of patients included in our study, we revealed that 16 patients harbored 14 novel *ROS1* fusion partners, all of whom had lung cancer. Of these, a novel *DCBLD1-ROS1* fusion was observed in three cases, while other fusions occurred once in six cases, and seven novel partners occurred in two retroperitoneal neoplasm patients (*FRK-ROS1*, *VGLL2-ROS1*), one gastric cancer patient (*ARHGEF11-ROS1*), one liver cancer patient (*REV3L-ROS1*), one liposarcoma patient (*EPHA7-ROS1*), one patient with epithelioid hemangioendothelioma (*NOTCH1-ROS1*), and one squamous cell carcinoma patient (*CARD11-ROS1*). These novel ROS*1* fusion partners should be further explored and may also be linked to promising therapies. For example, FPK is a Fyn-related kinase that functions as a tumor suppressor. It has been reported to inhibit glioma progression by suppressing ITGB1/FAK signaling. The effects of specific kinase inhibitors in *FRK-ROS1* fusion-positive cases should be explored further.

Variable genomic breakpoints have been identified in Chinese patients through GNS, most location was between exons 32 and 34, and exon 35was also a common. Most of canonical ROS1 fusions were sensitive to crizotinib, especially CD74-ROS1 fusion. Many novel uncommon ROS1 fusions have been found using NGS, most of which were reported to be sensitive to matched targeted therapy, similar to the canonical fusions (Hung et al., 2022). Clinical significance of some genomic breakpoints remained unclear. Simultaneously, more in-depth studies should be conducted to confirm and explore the mechanism underlying these fusions.

This study had numerous limitations. First, the response of the novel *ROS1* fusion types to the ROS1 inhibitors was not clear, and even the same *ROS1* fusion types with different fusion breakpoints of the partners or the *ROS1* gene should be collected, which will be beneficial for clinical therapy. In addition, the specific biological function of gene fusion has not been experimentally determined, and technical errors in NGS analyses cannot be completely excluded. Finally, although NGS is an established and powerful tool, there are barriers to its extensive application. It is expensive for patients and requires complicated equipment and skilled personnel to perform sequencing and subsequent bioinformatics analysis.

## Conclusion

In summary, this study was performed to detect *ROS1* fusion partners and *ROS1* fusion breakpoints in solid tumors of Chinese patients in the largest *ROS1* fusion cohort to date. Fourteen novel *ROS1* fusions were identified in the solid tumors of Chinese patients, and the *ROS1* breakpoint was located between exons 32 and 34 in many patients. Moreover, this study showed that NGS fusion assays can be used on plasma and tissue samples. NGS is a potent tool for reliably identifying novel *ROS1* fusions and for detecting molecular alterations.

## Data Availability

The data presented in the study are deposited in the Genome Sequence Archive for human (GSA-human) repository, accession number HRA003505, under the BioProject PRJCA013094.
