# Supplementary material for: Detection of ROS1 gene fusions using next-generation sequencing for patients with malignancy in China
Source: Front Cell Dev Biol. 2022 Dec 15;10:1035033. doi: 10.3389/fcell.2022.1035033 (PMC9798300; doi:10.3389/fcell.2022.1035033)
Supplement: Supplementary file 1 [file Table1.docx]

**Supplementary Table 1: Histologies of the 92 patients.**

| Cancer | Number | Age (Range) |
| --- | --- | --- |
| Lung cancer | 82 (89.1%) | 58 (28-82) |
| Retroperitoneal neoplasm | 2 (2.2%) | 50, 66 |
| Gastric cancer | 2 (2.2%) | 60, 69 |
| Colorectal cancer | 1 (1.1%) | 49 |
| Liver cancer | 1 (1.1%) | 60 |
| Liposarcoma | 1 (1.1%) | 54 |
| Schwannoma | 1 (1.1%) | 16 |
| Epithelioid hemangioendothelioma | 1 (1.1%) | 24 |
| Squamous cell carcinoma | 1 (1.1%) | 65 |
